# Household dairy production, dairy intake, and anthropometric outcomes in rural Bangladesh

**DOI:** 10.1016/j.foodpol.2023.102567

**Published:** 2023-11

**Authors:** M. Mehrab Bakhtiar, John Hoddinott

**Affiliations:** a International Food Policy Research Institute, USA; b Cornell University, USA

**Keywords:** Dairy cows, Dairy consumption, Child nutrition, Bangladesh

## Abstract

We assess whether ownership of dairy cows is associated with a greater likelihood of consuming dairy products and with child anthropometric status in rural Bangladesh. Consistent with the assumption of imperfectly functioning markets for dairy products, ownership of dairy cows increases the likelihood that a child 6–59 months consumes milk by 7.7 percentage points with no difference in this association between boys and girls. This association nearly doubles in magnitude when we consider households that own a dairy cow that produced milk in the last year. This result is robust to the controls we use and the way in which we measure dairy cow ownership. Even when we saturate our model with child, maternal, household, wealth, as well as village fixed effects, we retain an association between dairy cow ownership and height-for-age z scores (HAZ) that is meaningful in magnitude – 0.13 standard deviations – and statistically significant at the one percent level. For children in the 12–23.9 month age group, ownership of a dairy cow is associated with a 0.37 SD increase in HAZ and a reduction of 11.3 percentage points in stunting. There is no statistically significant association with weight-for-height or wasting. These associations do not differ between boys and girls.

## Highlights

• Using household survey data from rural Bangladesh, we examine whether ownership of dairy cows is associated with a greater likelihood of consuming dairy products and with child anthropometric status in rural Bangladesh.
• Consistent with the assumption of imperfectly functioning markets for dairy products, ownership of dairy cows increases the likelihood that a child 6–59 months consumes milk by 7.7 percentage points with no difference in this association between boys and girls. This association nearly doubles in magnitude when we consider households that own a dairy cow that produced milk in the last year.
• Dairy cow ownership is associated with an increase of 0.13 SD in height-for-age z scores (HAZ). For children in the 12–23.9 month age group, ownership of a dairy cow is associated with a 0.37 SD increase in HAZ and a reduction of 11.3 percentage points in stunting. There is no statistically significant association with weight-for-height or wasting.
• These associations do not differ between boys and girls. However, when dairy cows are owned solely by a female head or a woman who is the spouse of the head, there is suggestive evidence that the association between female ownership and HAZ is larger for boys than for girls.
• In rural Bangladesh, access to dairy products through ownership of dairy cattle is associated with both increased likelihood that infants and young children consume these nutrient dense foods and improved nutritional status. But because most rural children reside in homes where these dairy cows are not present, devising gender-sensitive ways of increasing access to these foods – for example, through increased local production and dairy value chain development - represents an important area for future intervention design and research.

## 1 Introduction

Improving the diet and nutritional status of infants and young children is of both intrinsic and instrumental value. It is of intrinsic value because healthy diets and good health are of value in their own right. They are of instrumental value because children who are better nourished in early life progress further in school, are more economically productive in adulthood and are less likely to be poor (Hoddinott, 2013). For these reasons, there is considerable interest in understanding what causes poor nutrition and how these can be addressed.

In the most general terms, poor nutritional status reflects poor diets and/or poor health status, each with factors that underlie these (Black, 2013). With respect to diets, Headey, Hirvonen, and Hoddinott (2018) document both biological and observational studies linking the consumption of animal source foods (ASFs) to improved nutritional outcomes in developing countries. One important ASF is dairy. Milk is (depending on fat content) energy-dense, protein rich and an excellent source of folate and calcium. It also contains B vitamins, vitamin K, iron, magnesium, phosphorous, and zinc. While other foods also contain many of these nutrients, cow’s milk appears to have two unique components that play a role in linear growth. First, as Van Den Hooven et al. (2015) note, milk contains calcium, phosphorus, and protein all of which are constituents of bone; adequate intake of these nutrients is needed for normal bone development. Other nutrients, including vitamin D, vitamin K, magnesium, zinc, and fluoride, are also involved in bone metabolism. Many of these – including vitamin K, magnesium, and zinc – are found in milk. It is increasingly well-understood that these nutrients act in synergistic ways. Vitamin D, for example, plays a role in the absorption of calcium; magnesium affects vitamin D and calcium metabolism (Van Den Hooven et al., 2015). Second, Insulin-Like Growth Factor-1 (IGF-1) is a hormone that promotes bone and tissue growth; specifically, it plays a role in bone growth by increasing the uptake of amino acids, which are then integrated into new proteins in bone tissue. It also has anabolic effects on growing bone tissue by stimulating chondrocytes in the epiphyseal plate (Hoppe, Mølgaard, & Michaelsen, 2006). Hoppe et al. (2006) argue that milk consumption increases the circulating concentrations of IGF-1, possibly through the presence of certain bioactive peptides or possibly through the presence of IGF-1 in cow’s milk. For these reasons, dairy consumption has the potential to improve child nutrition in early life, particularly in the period of 6–24 months of age when children are no longer exclusively breastfed and physical growth potential is high (Black et al., 2013, Shrimpton et al., 2001).

That said, access to dairy is problematic in hot, humid environments where the physical infrastructure needed for a perishable food’s value chain – specifically electricity and refrigeration – is underdeveloped. In such localities, physical access to dairy products – for example through ownership of a dairy cow – may be an important factor for children’s consumption of these foods and their nutritional status. Njuki et al. (2016) note that there are three pathways linking dairy cow ownership and children’s anthropometric outcomes: (a) a food production pathway whereby increased on-farm production of dairy products improve access to dairy; (b) an income pathway by which increased income from dairy sales is used to purchase food or non-food goods that improve anthropometric status; and (c) a women’s empowerment pathway where increased ownership of dairy cows or dairy products by women leads to improvements in the nutritional status of their children. Four studies (Choudhury and Headey, 2018, Hoddinott et al., 2015, Kabunga et al., 2017, Rawlins et al., 2014) show that dairy cow ownership improves linear growth as measured by HAZ or stunting. However, Mosites et al. (2016) show no such associations and a sixth, by Mosites et al. (2015) finds very small associations between livestock ownership and stunting in Ethiopia and Kenya, but not in Uganda.

At first glance, it is not clear what to make of these somewhat mixed findings which come from a limited number of countries (mostly Ethiopia, Kenya, and Uganda) and none use an experimental design. There use of different estimators and different control variables is one obvious challenge. A second is the fact that three of these studies focus on ownership of dairy cows with two of these showing statistically significant associations with child nutritional status. Others subsume dairy cow ownership within a broader category of “livestock” which also includes non-milk producing animals and thus would be expected to show weaker associations with child nutritional status. This distinction between milk-producing and non-milk producing animals is also central to the Choudhury and Headey (2018) study, which finds that only the former has a (large) and statistically significant association with HAZ. Next, note that many of these studies use a wide age range, typically 1–59 months. However, children less than 6 months should be exclusively breastfed and children who consume animal milk before age 12 m can suffer digestive problems (Fao, 2013). For older children, 24–59 months, growth trajectories are largely already established (Victora, De Onis, Hallal, Blössner, & Shrimpton, 2010) and so associations with linear growth for children in that age group may be attenuated. Lastly, as Hoddinott et al. (2015) emphasize, the direct effects of dairy cow ownership on children’s nutritional status will be more important in rural environments where there are significant market imperfections, i.e., where markets for dairy products are poorly developed. To the best of our knowledge, the only study that focuses on ownership of dairy cattle, on children 6–23 m, and accounts for market access is the paper by Hoddinott et al. (2015).

Given the mixed findings in the extant literature, in this paper we examine associations between ownership of dairy cows, children’s consumption of dairy products and their child anthropometric status using data from rural Bangladesh. Because we report associations, we are careful to assess whether our results are robust to omitted variable bias. In addition to providing new evidence on this topic, we extend this literature in two ways: first, by assessing robustness of these associations to measurement of dairy cow ownership; and second, by examining whether the identity of the owner of these animals affects these associations.

The paper is structured as follows. Section 2 describes our methods and data. Section 3 provides descriptive statistics on dairy production in our sample as well as the characteristics of the children used in our analysis and the households that they reside in. Section 4 provides results on dairy consumption and child anthropometric status.

## 2 Methods and data

### 2.1 Methods

We build on the approaches found in Hoddinott et al. (2015) and Choudhury and Headey (2018). Adapting the agricultural household model of Singh, Squire, and Strauss (1986), households have a welfare function that includes child nutritional status, nutrient intake and other variables that affect household welfare. This is maximized subject to a series of constraints including a nutrition production function (linking inputs to the determinants of child nutritional status), an agricultural production function, and a full income (time and budget constraint). Under the assumption that the market for dairy products is missing, assets that produce milk, namely dairy cows, appear as arguments in the reduced form demand functions for child nutritional status and a child’s milk consumption (De Janvry et al., 1991, Hoddinott et al., 2015). Missing markets have been shown to be a reasonable assumption for a highly perishable good that is produced in a tropical climate where there is little access to refrigeration and value chains are underdeveloped. In addition to dairy cows, our models include the following covariates: child characteristics, age and sex (reflecting the idea that for biological reasons, the nutrition production function varies by age and sex); maternal characteristics that either directly affect nutritional status through a biological channel (maternal height) or do so through their impact on care giving practices (maternal knowledge and age, the latter proxying for maternal experience); household demographic and wealth characteristics (size, which may create economies of scale in certain aspects of household consumption; household head, age education, and sex which capture both the life cycle position of the household and potentially income generating capacity; land ownership, ownership of livestock measured in Tropical Livestock Units (TLU), and a household asset index).^1^ We also control for locality factors such as access to markets and to health facilities and the health environment in which the child lives. We estimate the following model:

$$Y_{c,H,M,Y,V}=\beta_{\mathrm{Cow}}Cow_{H,M,Y}+\beta_{C}Child_{C,H,M,Y}+\beta_{M}Mother_{H,M,Y}+\beta_{H}Household_{H,M,Y}+\beta_{M,Y}MonthYear_{M,Y}+\beta_{V}Village_{V}+\varepsilon_{C,H,M,Y,V}$$

Where Y_c, H, M, Y, V_ is our outcome variable for child c living in household H and village V, observed in month M of year Y. Our coefficient of interest is β_COW_, showing how measures of dairy cow ownership are associated with our outcome. We control for a vector of child (Child), maternal (Mother) and household and wealth (Household) characteristics; these vectors are denoted in bold. We include village fixed effects. These capture time-invariant locality factors (for example, access to markets) associated with our outcome variables. Coefficients associated with these vectors are denoted as **β** and the disturbance term is ε_C, H, M, Y, V_ We use least squares estimates; where the outcome variable is dichotomous, we use a linear probability model. In estimates where we do not control for village fixed effects, standard errors are clustered at the sampling unit, the village.

### 2.2 Data

We use data from three rounds of the Bangladesh Integrated Household Survey (BIHS). The BIHS is a cluster-based multi-topic longitudinal survey designed to be representative of households living in the rural areas of each the seven administrative divisions^2^ of the country: Barisal, Chittagong, Dhaka, Khulna, Rajshahi, Rangpur, and Sylhet (Ahmed, 2013). The survey has been fielded in 2011–2012, 2015, and 2018–2019. A strength of the BIHS is that it contains considerable detail on dairy cow ownership and milk production, including questions on: number of dairy cows owned, who within the household owns the animals, whether the cows produce milk, the amount of milk produced, and where and when milk is sold. Questions were administered to the household head and his spouse and so there are some cases where a mother provides data on more than one child.

For our assessment of the associations between dairy cow ownership and child diet, we utilize 24-hour dietary recall data. These data were collected by female enumerators who interviewed mothers about all foods consumed the previous day. Mothers were asked to list the foods, by meal, which were consumed (the household’s “menu”), the ingredients used to prepare these, and their raw and cooked weights. We use these data to determine if a child consumed a dairy product such as milk, yogurt, or cheese at some point during the previous day.

We assess associations with nutritional status by calculating height-for-age z-score (HAZ).^3^ The z-score measure is calculated using the WHO child growth standards (WHO (World Health Organization) (2006) (World Health Organization), 2006). For HAZ, a value of −1 indicates that, given sex and age, a child’s height is one standard deviation below the median child in her age/sex group reference group. HAZ is a measure of chronic undernutrition. It can be thought of as a summary indicator of many factors that influence growth and development. In addition, we also use weight-for-height z-scores (WHZ) as an additional outcome. These assess a child’s weight given her height relative to the WHO reference population. Low WHZ is an indicator of acute undernutrition, reflecting recent illness, inadequate nutrients, or both. We assess the robustness of our results on HAZ by considering stunting, which equals one if the child has a HAZ less than −2 and the robustness of our WHZ results by considering wasting, which equals one if the child has a WHZ less than −2.

## 3 Descriptive statistics

### 3.1 Dairy cow ownership and milk production

We begin with some descriptive data on the ownership and productivity of milk cows.^4^ At the time of the first survey in November 2011, 25.8 percent of all households in the BIHS sample owned one or more cows (Table 1). Herd sizes were small with only 5.5 percent of households owning three or more cows. With some variation over time, these broad patterns persist over time with the percentage of households with one or more cows, rising only slightly to 28.0 percent by 2018. The average household in our sample owns between 0.37 (2015) to 0.55 (2018) cows; conditional on owning one or more cows, mean ownership of cows was 1.95 in 2018. Consumption and sale of milk is the reason given by approximately 90 percent of households for owning cows. Not all cows produce milk and so only 16.5 to 18.4 (depending on the survey round) of households had cows producing milk at some point in the last 12 months. Each BIHS survey asked respondents to estimate how much milk was produced in the last 12 months. Conditional on any production, mean production of milk ranged from 0.66 to 0.81 L per day. However, as Supplementary Fig. S1 shows for 2018, distribution of production is skewed with the median producing household producing less than 0.5 L per day.^5^ Between 29.2 and 40.5 percent of households with a cow report selling milk in the 12 months preceding the survey. In all rounds, between 56 and 59 percent of these sales were made at the farmgate with another 23 to 27 percent made in markets in the village where the household resides. Generally, cows are owned by men resident in the household. Joint ownership by men and women is also common with 18.4 (2018) to 33.8 (2015) percent of households reporting this. Sole ownership by women was uncommon, nine to 12 percent.

**Table 1:** Ownership of dairy cows, by round.

	Year	2011	2015	2018
		Percent		
Distribution of ownership	0 cows	74.2	79.0	72.0
	1	11.2	10.9	11.5
	2	9.1	6.3	10.4
	3 or more	5.5	3.8	6.1
		Number		
Number owned	Mean	0.50	0.37	0.55
	Mean conditional on owning 1 or more	1.94	1.77	1.95
		Percent		
Purpose of ownership	Milk consumption only	1.6	0.7	1.0
	Sale of milk only	6.3	8.4	9.5
	Consumption and sale of milk	92.1	90.9	89.5
		Percent		
Milk production	Produced any milk in last 12 months	18.4	16.5	18.3
		Liters per day		
	Mean production conditional on producing any milk	0.69	0.81	0.66
		Percent		
Sales	Made any sale of milk, conditional on ownership	34.8	40.5	29.2
Conditional on making sales, where sold				
	Farmgate (home)	59.0	56.1	59.4
	In market within village	23.2	27.7	27.4
	In market outside village	17.8	16.2	13.2
Who owns	Sole male	58.9	46.7	59.6
	Joint male/female	23.0	33.8	18.4
	Sole female	9.4	10.6	12.4
	Other	8.7	8.9	9.6

Notes: Source. BIHS rounds fielded in 2011, 2015 and 2018.

Asking about milk production over the last 12 months is demanding in terms of respondent recall. In the 2018 round, respondents were also asked about milk production the previous day. Conditional on any milk production, mean daily production was 2.1 L with most of this (85 percent) produced in the morning and a much smaller amount, 15 percent, produced in the evening. However, only 34 percent of households with dairy cows reported any milk production the previous day. Across all households with a dairy cow, mean daily production was 0.72 L, similar to the 0.66 L per day statistic based on the 12-month recall. Amongst those who sell milk, most sold either only in the morning (79.8 percent) or in the morning and evening (11.6 percent).

In all rounds, the BIHS survey instrument included questions on ownership of dairy cows 12 months prior to the interview. We can use these data to assess the extent to which holdings of dairy cows change over time. As Supplementary Table S1 shows, households that did not own any dairy cows 12 months ago were highly unlikely to report that they had acquired a dairy cow – in all three rounds, entry into ownership was around two or three percent. Those households who had one dairy cow a year ago, were most likely to continue to have one cow at the time of the survey (61 – 68 percent, depending on the survey round) and a majority (around 54 percent) who had two cows a year ago were likely to continue to have two cows when interviewed. Put differently, for most households, ownership (and non-ownership) is static over time. Only between a quarter and a third of households with one or two cows report adding to their herd over the 12 months preceding the survey, with a smaller fraction reporting that they reduced their holdings over that time. A small percentage of households (four to seven percent) with only one cow reported no longer holding dairy cows at the time of the survey. The 2018 survey round included additional questions on dairy cows and production. This showed that among households owning any dairy cattle, around 80 percent were low milk yielding local varieties. Most (79 %) are not vaccinated and are fed through a mix of grazing and consuming rice straw. Few households belong to a milk marketing cooperative.

### 3.2 Child characteristics

Table 2 describes the children in our sample. We use data on children aged 6–59 months. Children under the age of six months should be exclusively breastfed and thus are excluded from our sample. We also exclude children with invalid height-for-age z scores, those greater than six or less than −6 (WHO (World Health Organization) (2006), 2006). Thus, the sample consists of 6,086 child observations equally distributed across all three survey rounds. There are slightly fewer girls than boys with girls comprising 48.2 percent of the sample. Mean child age is 32.4 months with little difference across survey rounds. One-third of the sample is between 6 and 24 months.

**Table 2:** Child characteristics.

	Unit	2011	2015	2018	All rounds	Girls	Boys
Sample size	Number	2105	2001	1980	6086	2932	3154
Girls	Percent	50.4	46.5	47.5	48.2	–	–
Mean age	Months (m)	31.8	32.7	32.7	32.4	32.6	32.2
	SD	15.3	15.4	15.6	15.4	15.5	15.4
Children 6–23m	Percent	34.8	31.9	33.3	33.4	33.4	33.4
Children > 23m	Percent	65.2	68.1	66.7	66.6	66.6	66.6
Child consumed dairy product (milk, yoghurt, cheese) during previous day	Percent	21.1	31.3	33.6	28.6	28.1	29.0
Child consumed dairy product, child 6–11.9m	Percent	33.2	37.6	35.6	35.4	32.6	38.0
Child consumed dairy product, child 12–17.9m	Percent	20.3	33.2	27.7	26.8	25.7	27.8
Child consumed dairy product, child 18–23.9m	Percent	25.3	39.2	43.7	35.9	37.7	34.2
Child consumed dairy product, child > 23.9m	Percent	18.9	29.2	32.9	26.9	26.6	27.2
Height for age z score	Mean	−1.91	−1.69	−1.51	−1.71	−1.69	−1.72
	SD	1.44	1.29	1.23	1.34	1.29	1.38
Stunted	Percent	49.2	40.4	34.2	41.4	40.7	42.1
	SD	0.50	0.49	0.47	0.49	0.49	0.49
Weight for height z score	Mean	−0.74	−0.96	−0.66	−0.79	−0.79	−0.79
	SD	1.19	1.20	1.14	1.18	1.14	1.23
Wasted	Percent	11.8	16.5	9.6	12.7	12.3	13.0
	SD	0.32	0.37	0.29	0.33	0.33	0.34

Notes: See Table 1.

Across the full sample, 28.6 percent of all children consumed a dairy product (milk, yogurt, cheese) during the previous day. There is no difference between the likelihood that boys and girls consume milk. The percentage of children consuming milk, however, rises over time from 21.1 percent in 2011 to 33.6 percent in 2018. This change is particularly pronounced for children aged 12–17.9 months and 18–23.9 months.

Mean height-for-age z scores (HAZ) across all three rounds is −1.71. However, this masks a marked improvement over time. Mean HAZ increases from −1.91 in 2011 to −1.69 in 2015 and −1.51 in 2018. Concomitantly, the percentage of children considered stunted (i.e. with HAZ < -2) falls from 49.2 percent in 2011 to 34.2 percent in 2018.

### 3.3 Control variables

Table 3 summarizes the maternal and household characteristics used in our regressions. Mothers are, on average, 27 years old with 5.4 grades of schooling. Most, 65 percent, are spouses of the household head but there are also mothers who are daughters-in-law (17 percent) or, in a few cases (two percent), daughters of the head. The average household has 5.2 members. The average age of the household head is 39.8 years. These heads have completed 3.8 grades of schooling and 17 percent are women. On average, households own 55 decimals of land (0.55 acres) and 0.81 TLU.

**Table 3:** Maternal and household characteristics.

	Mean	Standard Deviation
Maternal characteristics		
Age	27.3	5.7
Schooling	5.4	3.6
Height	150.9	5.6
Mother’s relationship to male head		
Spouse	0.77	0.42
Daughter	0.04	0.18
Daughter-in-law	0.17	0.37
Other	0.02	0.15
Household characteristics		
Household size	5.2	1.9
Head is female	0.17	0.37
Head, age	39.8	13.3
Head, grades of schooling	3.8	3.8
Amount of land owned, decimals (hundredths of acres)	55.4	121.3
Tropical Livestock Units	0.81	1.57
Wealth index	−0.06	1.96

Notes: See Table 1.

## 4 Results

### 4.1 Dairy consumption

We begin with the first column of Table 4. Here, we only include a dichotomous indicator equaling one if the household owned any dairy cows, zero otherwise; this is equivalent to assuming that all β’s in our estimated equation are equal to zero except for β_Cow_. The parameter estimate for β_Cow_ is 0.102, indicating that ownership of cows is associated with a 10.2 percentage point increase in the likelihood that the child aged 6–59 months consumed a dairy product (milk, yogurt, cheese) during the previous day. The association is significant at the one percent level. In column (2), we add child characteristics, sex, and dummy variables for age in months. Columns (3) and (4) add, sequentially survey month and year fixed effects and then village fixed effects. The association remains positive and significant and similar in magnitude, 0.094 in column (3) and 0.133 in column (4). When we fully saturate the model with child, mother, and household characteristics (including household wealth), and we control for survey month and year fixed effects, we find that ownership of a dairy cow is associated with a 7.7 percent increase in the likelihood that the child consumed a dairy product the previous day; this association is statistically significant at the one percent level. We get similar magnitudes when we disaggregate by child sex.

**Table 4:** Basic results, consumption of dairy products.

	(1)	(2)	(3)	(4)	(5)	(6)	(7)
Outcome	Child consumed dairy product (milk, yoghurt, cheese) during previous day						
Household currently owns dairy cow	0.102***	0.100***	0.094***	0.133***	0.120***	0.116***	0.077***
	(0.02)	(0.02)	(0.02)	(0.01)	(0.01)	(0.01)	(0.01)
Child characteristics	No	Yes	Yes	Yes	Yes	Yes	Yes
Survey month and year fixed effects	No	No	Yes	Yes	Yes	Yes	Yes
Village fixed effects	No	No	No	Yes	Yes	Yes	Yes
Mother characteristics	No	No	No	No	Yes	Yes	Yes
Household characteristics	No	No	No	No	No	Yes	Yes
Wealth characteristics	No	No	No	No	No	No	Yes
Observations	5,824	5,824	5,824	5,824	5,824	5,824	5,824
R-squared	0.010	0.027	0.045	0.050	0.065	0.068	0.085
Number of villages	275	275	275	275	275	275	275

Notes: (a) Models estimated using linear probability models. (b) Child characteristics: Sex, dummy variables for age in months. (c) Mother characteristics: Log age, grades of schooling, log height, dummy variable = 1 if mother is spouse of head, dummy variable = 1 if mother is daughter of head, dummy variable = 1 if mother is daughter-in-law of head, dummy variable = 1 if mother is other relation to head. (d) Household characteristics: Household size, sex of household head, age of household head, grades of schooling of household head. (e) Wealth characteristics: quintile of land ownership, household asset index (PCA) and Tropical Livestock Units (TLU). (f) Standard errors in parentheses. Standard errors are clustered at village level in columns (1)-(3); calculated accounting for village fixed effects in columns (4)-(8). (g) * significant at the 10 percent level; ** significant at the 5 percent level; *** significant at the 1 percent level.

Next, we assess whether our results are sensitive to how we measure access to dairy (Table 5). A simple robustness check is to replace the dummy variable for cow ownership with the number of dairy cows owned. As column (2) shows, this also produces a positive and statistically significant association. As a further check, we created dummy variables for the following categories: a household owns one dairy cow; owns two; owns three; and owns four or more (only two percent of children live in households with four or more dairy cows). There is some suggestion of non-linearities in the relationship with the respective coefficients being 0.050, 0.093, 0.150 and 0.123 respectively (all significant at the 5 % level). One explanation for this pattern is that with more than one cow, the likelihood that at least one is lactating increases. Consistent with this idea, we note that Choudhury and Headey (2018) argue that the association between the dummy variable for cow ownership and dairy consumption will be attenuated because dairy cows do not produce milk continuously. When we compare the results shown in Table 6, column (1) to Table 5, column (3), we see evidence of this. The coefficient for ownership of dairy cows that produced milk in the last 12 months doubles in magnitude (0.141) and statistically significant at the one percent level. By contrast, the coefficient on ownership of dairy cows not producing milk is essentially zero and not statistically significant. Lastly, in column (4), we exploit the fact that the BIHS (unlike the Ethiopian data used by Hoddinott et al., 2015) contains information on household reports of how much milk was produced in the last 12 months. Recall error will bias this association downwards but with that caveat, it is noteworthy that even with a full set of child, maternal, household controls as well as controls for survey round and village fixed effects, we find a statistically significant association between daily production (in liters) and dairy consumption with every liter of milk produced on farm increasing the likelihood that a child consumes dairy products by 7.8 percentage points.^6^

**Table 5:** Consumption of dairy products, robustness checks.

	(1)	(2)	(3)	(4)
Outcome	Child consumed dairy product during previous day			
Household currently owns dairy cow	0.077***	–	–	–
	(0.02)			
Number of cows currently owned	–	0.033***	–	–
		(0.01)		
Household currently owns dairy cow that produced milk in last year	–	–	0.141***	–
			(0.02)	
Household currently owns dairy cow that did not produce milk in last year	–	–	− 0.010	–
			(0.03)	
Average daily production of milk in last 12 months	–	–	–	0.078***
				(0.01)
Child characteristics	Yes	Yes	Yes	Yes
Survey month and year fixed effects	Yes	Yes	Yes	Yes
Village fixed effects	Yes	Yes	Yes	Yes
Mother characteristics	Yes	Yes	Yes	Yes
Household characteristics	Yes	Yes	Yes	Yes
Wealth characteristics	Yes	Yes	Yes	Yes
Observations	5,824	5,824	5,824	5,824
R-squared	0.085	0.084	0.091	0.087
Number of villages	275	275	275	275

Notes: All estimates control for child, mother, household, and wealth characteristics as well as survey month and year and village fixed effects. For additional notes, see Table 5.

**Table 6:** Basic results, associations with length/height-for-age z score.

	(1)	(2)	(3)	(4)	(5)	(6)	(7)
Outcome	Length/height-for-age Z-score						
Household currently owns dairy cow	0.181***	0.182***	0.160***	0.211***	0.150***	0.152***	0.127***
	(0.04)	(0.04)	(0.04)	(0.04)	(0.04)	(0.04)	(0.05)
Child characteristics	No	Yes	Yes	Yes	Yes	Yes	Yes
Survey month and year fixed effects	No	No	Yes	Yes	Yes	Yes	Yes
Village fixed effects	No	No	No	Yes	Yes	Yes	Yes
Mother characteristics	No	No	No	No	Yes	Yes	Yes
Household characteristics	No	No	No	No	No	Yes	Yes
Wealth characteristics	No	No	No	No	No	No	Yes
Observations	6,086	6,086	6,086	6,086	6,086	6,086	6,086
R-squared	0.003	0.047	0.063	0.068	0.125	0.126	0.131
Number of villages	–	–	–	275	275	275	275

Notes: (a) Sample consists of children 6–59 m with HAZ > -6 and HAZ < 6. (b) Child characteristics: Sex, dummy variables for age in months. (c) Mother characteristics: Log age, grades of schooling, log height, dummy variable = 1 if mother is spouse of head, dummy variable = 1 if mother is daughter of head, dummy variable = 1 if mother is daughter-in-law of head, dummy variable = 1 if mother is other relation to head. (d) Household characteristics: Household size, sex of household head, age of household head. (e) Wealth characteristics: Grades of schooling household head, quintile of land ownership; (f) Standard errors in parentheses. Standard errors are clustered at village level in columns (1)-(3); calculated accounting for village fixed effects in columns (4)-(8). (g) * significant at the 10 percent level; ** significant at the 5 percent level; *** significant at the 1 percent level.

### 4.2 Anthropometry

We consider children aged 6–59 months, excluding observations with HAZ > 6 and HAZ < -6 as these are biologically implausible (WHO (World Health Organization) (2006), 2006). In the first column of Table 6, we only include an indicator variable equaling whether the household owned any dairy cows, zero otherwise; assuming that all β’s in our estimated equation are equal to zero except for β_COW_. The parameter estimate is 0.181, indicating that ownership of cows is associated with an increase of 0.18 standard deviations in HAZ. The association is significant at the one percent level. In column (2), we add child characteristics, sex, and dummy variables for age in months and in columns (3) and (4) add, sequentially survey month and year fixed effects and then village fixed effects. The association remains positive and significant and similar in magnitude, 0.160 in column (3) and 0.211 in column (4). Adding maternal characteristics results in the association falling in magnitude to 0.150 but remains statistically significant at the one percent level (column 5). Adding household characteristics (column 6). Adding measures of household wealth and income causes the association to fall slightly (column 7). Summarizing, even when we saturate our model with child, maternal, household, wealth, survey round, survey month controls as well as village fixed effects, we retain an association between dairy cow ownership that is meaningful in magnitude, 0.127 standard deviations, and statistically significant at the one percent level.

In Table 7, we extend our results by conducting additional robustness checks and extensions using the specification that controls for child, maternal, household, wealth, survey round, survey month controls as well as village fixed effects. In column (1), we replace the dummy variable for ownership of any dairy cows with the number of dairy cows owned. This shows a positive relationship with each additional dairy cow associated with a 0.059 standard deviation increase in HAZ, statistically significant at the one percent level. In column (2), we replace the outcome variable with a dummy variable equaling one if the child was stunted, zero otherwise. Ownership of a dairy cow is associated with a 4.3 percentage point reduction in the likelihood that a child is stunted. When we replace ownership of any dairy cows with ownership of dairy cows producing milk (columns 3 and 4), we see that milk producing dairy cows is associated with an increase of 0.136 SD in HAZ and a reduction of 4.7 percentage points in the likelihood that a child is stunted. The level of daily milk production is associated with an increase in HAZ; every additional liter of production is associated with an increase of 0.112 SD (column 5). Milk production is associated with a lower likelihood of being stunted, though this association is imprecisely measured (column 6). In columns (7) and (8), we consider measures of acute nutritional status, weight for length/height z-scores (column 7) and whether the child is wasted (column 8). In both specifications, we find no association with current ownership of a dairy cow.

**Table 7:** Associations of cow ownership: Robustness and other anthropometric outcomes.

	(1)	(2)	(3)	(4)	(5)	(6)	(7)	(8)
Outcome	Length/ height-for-age Z-score	Stunted	Length/ height-for-age Z-score	Stunted	Length/ height-for-age Z-score	Stunted	Weight-for-length/height Z-score	Wasted
Number of dairy cows currently owned	0.059***	–					–	–
	(0.02)							
Household owns dairy cow	–	−0.043***					−0.051	0.018
		(0.02)					(0.04)	(0.01)
Household currently owns dairy cow that produced milk in last year			0.136***	−0.047***				
			(0.05)	(0.02)				
Household currently owns dairy cow that did not produce milk in last year			0.075	−0.012				
			(0.09)	(0.03)				
Average daily production of milk in last 12 months					0.112***	−0.007		
					(0.04)	(0.01)		
Child characteristics	Yes	Yes	Yes	Yes	Yes	Yes	Yes	Yes
Survey month and year fixed effects	Yes	Yes	Yes	Yes	Yes	Yes	Yes	Yes
Village fixed effects	Yes	Yes	Yes	Yes	Yes	Yes	Yes	Yes
Mother characteristics	Yes	Yes	Yes	Yes	Yes	Yes	Yes	Yes
Household characteristics	Yes	Yes	Yes	Yes	Yes	Yes	Yes	Yes
Wealth characteristics	Yes	Yes	Yes	Yes	Yes	Yes	Yes	Yes
Observations	6,086	6,086	6,086	6,086	6,086	6,086	6,078	6,078
R-squared	0.131	0.106	0.131	0.106	0.131	0.105	0.055	0.029
Number of villages	275	275	275	275	275	275	275	275

Notes: Stunting and wasting estimates are linear probability models. For other notes, see Table 7.

Next, we assess the sensitivity of these results to the age of the child. As noted in Choudhry and Headey (2018), most growth faltering occurs between 0 and 24 months. However, children should be exclusively breastfed from birth to six months and consumption of cow’s milk before the age of one year is discouraged as children’s immature digestive systems cannot digest it well. Beyond two years, the period of rapid growth velocity ends. Table 8 disaggregates our HAZ results, first by six-month increments: 6–11.9 months; 12–17.9 months; and 18–23.9 months. We estimate these samples using our full set of child, maternal, household, wealth, survey round, survey month controls and village fixed effects. As expected, there is no association between dairy cow ownership and HAZ for children 6–11.9 months and the magnitude of this non-significant association is smaller, 0.08, than for the whole sample. The magnitude of the association for children 12–17.9 months and 18–23.9 months is much larger, around 0.30 for both, but imprecisely measured. However, note that these disaggregated samples are much smaller in size. When we combine these into a sample of children 12–23.9 months (column 4), we find that ownership of a dairy cow is associated with a 0.375 SD increase in HAZ or, as column (5) shows, a reduction of 11.3 percentage points in stunting. By contrast, as expected, for children 24–59.9 months, the association between current ownership and HAZ is small, 0.082, and not statistically significant.

**Table 8:** Associations of cow ownership with HAZ /stunting, by child age and sex.

	(1)	(2)	(3)	(4)	(5)	(6)	(7)	(8)
	Disaggregation							
	Age 6–11.9 months	Age 12–17.9 months	Age 18–23.9 months	Age 12–23.9 months	Age 12–23.9 months	Age 24–59 months	Girls: Age 12–23.9 months	Boys: Age 12–23.9 months
Outcome	Length/height-for-age Z-score				Stunted	Length/height-for-age Z-score		
Household currently owns dairy cow	0.065	0.325	0.287	0.375***	−0.113***	0.082	0.349*	0.291
	(0.2)	(0.17)	(0.21)	(0.12)	(0.040)	(0.06)	(0.18)	(0.21)
Child characteristics	Yes	Yes	Yes	Yes	Yes	Yes	Yes	Yes
Survey month and year fixed effects	Yes	Yes	Yes	Yes	Yes	Yes	Yes	Yes
Village fixed effects	Yes	Yes	Yes	Yes	Yes	Yes	Yes	Yes
Mother characteristics	Yes	Yes	Yes	Yes	Yes	Yes	Yes	Yes
Household characteristics	Yes	Yes	Yes	Yes	Yes	Yes	Yes	Yes
Wealth characteristics	Yes	Yes	Yes	Yes	Yes	Yes	Yes	Yes
Observations	691	758	570	1,328	1,328	4,055	650	678
R-squared	0.164	0.121	0.23	0.112	0.1	0.121	0.13	0.168
Number of villages	252	257	237	272	272	275	248	246

Notes: See Table 7, Table 8.

Table 8 also considers a second disaggregation, by child sex. Given the results described in the previous paragraph, we focus on the age group 12–23.9 months. Columns (7) and (8) show that the association between dairy cow ownership and HAZ is nearly identical for girls (0.349) and boys (0.291).^7^

As noted, a helpful feature of the BIHS is that it identifies the owner of the dairy cow. Table 9 assesses whether associations with HAZ differ by owner of the animal, focusing (given the results shown in Table 9) on children 12–23.9 months. The first column disaggregates by owner. An animal owned solely by the male head is associated with a 0.378 SD increase in HAZ for children in this age group. (Note that these regressions also control for child sex and sex of the household head.) When the animal is owned solely by a female household head or spouse of head, the association is larger, but we cannot reject the null hypothesis that it differs from the coefficient for sole male ownership. The second column interacts these ownership variables with child sex. There does not appear to be a difference in association between male ownership of a dairy cow and boys’ and girls’ HAZ as the interaction term is small in magnitude and not statistically significant. However, when dairy cows are owned solely by a female head or a woman who is the spouse of the head, the association between dairy cow ownership and boys’ HAZ (given by the coefficient for “solely by female head or spouse of head”) is large, 1.02, and statistically significant. By contrast, the coefficient for the interaction term (“solely by female head or spouse of head and child is female”), is large in magnitude but negative and imprecisely measured. This suggests that the association between female ownership and HAZ is larger for boys than for girls, but we caveat that there are only a small number of observations where the dairy animals are owned by female head or spouse of head and the child is a boy (approximately two percent of the sample).

**Table 9:** Associations of cow ownership with HAZ for children 12–23.9 m, by owner of cow.

	(1)	(2)
Outcome	Length/height-for-age Z-score	
Household currently has dairy cow owned:		
Solely by male head	0.378**	0.407**
	(0.15)	(0.19)
Solely by female head or spouse of head	0.591**	1.028**
	(0.27)	(0.40)
Jointly owned by male head and spouse	0.250	0.448*
	(0.19)	(0.24)
By other household members	0.428	0.548
	(0.28)	(0.40)
Solely by male head *and* child is female	–	−0.036
		(0.25)
Solely by female head or spouse of head *and* child is female	–	−0.810
		(0.53)
Jointly owned by male head and spouse *and* child is female	–	−0.421
		(0.36)
By other household members *and* child is female	–	−0.226
		(0.55)
Child characteristics	Yes	Yes
Survey month and year fixed effects	Yes	Yes
Village fixed effects	Yes	Yes
Mother characteristics	Yes	Yes
Household characteristics	Yes	Yes
Wealth characteristics	Yes	Yes
Observations	1,328	1,328
R-squared	0.113	0.117
Number of villages	272	272

Notes: See Table 7.

## 5 Conclusion and policy implications

The profound impact of diet and nutrition on the growth, development, and long-term health outcomes of infants and young children is well-documented in the literature. A critical question that arises, then, is how to optimize nutrition during this crucial period, particularly in low-income contexts where malnutrition is prevalent. In this paper, we address this question by focusing on the role of dairy consumption in improving the nutritional status of infants and young children in a low-income country context, rural Bangladesh.

We begin by summarizing our main results. Consistent with the assumption of imperfectly functioning markets for dairy products, across our full sample, ownership of dairy cows increases the likelihood that a child consumes milk by 7.7 percentage points with no difference in this association between boys and girls. This association nearly doubles in magnitude when we consider households that own a dairy cow that produced milk in the last year. This result is robust to the controls we use and the way in which we measure dairy cow ownership.

Across our full sample, even when we saturate our model with child, maternal, household, wealth, survey round, survey month controls as well as village fixed effects, we retain an association between dairy cow ownership and HAZ that is meaningful in magnitude – 0.13 standard deviations - and statistically significant at the one percent level. For children in the 12–23.9 month age group, ownership of a dairy cow is associated with a 0.37 SD increase in HAZ and a reduction of 11.3 percentage points in stunting. There is no statistically significant association with WHZ or wasting. These associations do not differ between boys and girls. There does not appear to be a difference in association between male ownership of a dairy cow and boys’ and girls’ HAZ.

We stress that these results are associations and should not be regarded as causal. While we control for a wide range of confounding factors, the decision to acquire dairy animals may, in part, reflect household preferences for milk. The decision by specific household members to begin raising dairy cows may reflect these preferences but also intra-household decision-making processes that are correlated with child diet and child nutritional status.

As noted in Hoddinott et al. (2015), these results do not imply that dairy cow ownership should become universal in rural Bangladesh or elsewhere. Rather, they imply that physical availability of milk products increases the likelihood that young children will consume these foods. This matters because dairy consumption is associated with improved children’s anthropometric status. What then are the policy implications? We note the following.

First, productivity of the dairy cows owned by rural Bangladeshi households (in the national BIHS sample) is low, a result also found in other studies of the dairy sector in Bangladesh (Datta, Haider, & Ghosh, 2019). One reason for this is the absence of high-yielding dairy cow breeds in Bangladesh. As most of the produced milk comes from small or marginal farmers who rear low-yielding local breeds, this bottleneck in the supply chain is slowing down the growth in the sector. To put it into context, an average local breed cattle’s yield is around one-twentieth of that of a crossbreed cattle. Thus, policies that encourage the access to and adoption of high-yielding breeds may lead to higher productivity. The success of such policies, however, may depend on increased availability of credit and complementary inputs such as improved access to veterinary services and improved animal feeds (Janssen & Swinnen, 2019).

Second, increased productivity will be of limited value if production in excess of home consumption cannot be readily marketed. But developing dairy value chains is challenging.

Not only do they need robust and reliable infrastructure– refrigeration and electricity – they must also address concerns over product adulteration and food safety (Janssen & Swinnen, 2019). Experiences elsewhere in South Asia (Morgan, 2009), that have found productivity gains in dairy production including the use of dairy cooperatives such as India’s experience with Operation Flood, are likely to be instructive.^8^

Third, as Mosites et al. (2015) and Penakalapati et al. (2017) both note, livestock ownership may increase children’s exposure to fecal material or zoonotic pathogens. The resulting illnesses, such as diarrhea, affect nutritional status in the short term by increasing metabolic demands and/or decreasing appetite and/or causing inflammatory patterns that reduce the absorption of nutrients. Where this happens repeatedly, linear growth may be reduced. Fourth, larger herds require more labor; where this provided by women, the time required to care for animals may conflict with time needed for childcare (Njuki et al., 2016). Finally, as Njuki et al. (2016) note, as home-based milk production rises in low-income contexts, milk may be increasingly sold. The revenues obtained may be controlled by male household members, who use these funds to purchase goods that do not directly affect children’s nutritional status. All these considerations might limit the potentially beneficial impacts of increased dairy production.

Our research indicates that in rural Bangladesh, access to dairy products through ownership of dairy cattle is associated with both increased likelihood that infants and young children consume these nutrient dense foods and improved nutritional status. But because most rural children reside in homes where these dairy cows are not present, devising gender-sensitive ways of increasing access to these foods – for example, through increased local production and dairy value chain development - represents an important area for future intervention design and research.

## CRediT authorship contribution statement

**M. Mehrab Bakhtiar:** Conceptualization, Investigation, Software, Formal analysis, Writing – original draft, Writing – review & editing, Methodology. **John Hoddinott:** Conceptualization, Investigation, Software, Formal analysis, Writing – original draft, Writing – review & editing, Methodology.

## Declaration of Competing Interest

The authors declare that they have no known competing financial interests or personal relationships that could have appeared to influence the work reported in this paper.

## Footnotes

1 The household asset index is constructed using a principal component analysis (PCA) on a set of assets common to all the survey rounds, excluding cash. For each asset, a binary variable is created, where 1 indicates the household owns the asset and 0 indicates otherwise. Then for each round, all consumer durable assets are inputted into the principal component analysis to construct a PCA score. The list of consumer durable assets collected in all survey rounds include trunk, buckets, stove, metal cooking pots, bed, cabinet, table/chair, hukka, electric fan, electric iron, radio, audio cassette, wall clock/watch, TV (black/white), TV (color), jewelry (gold/silver), sewing machine, bike, rickshaw, van, boat, engine boat, motorcycle, mobile phone set, land phone set, wooden rice husking pedal (dheki), grinder (jata), long knife (randa), saw, hammer, ring well (patkoa), fishing net, spade (Kodal), axe (Kural), shovel (belcha), shovel, sickle (daa), horse, mule, donkey, solar energy panel and electricity generator/ Integrated Power System (IPS).

2 In 2015, Mymensingh Division was created from Dhaka Division, becoming the eighth administrative division of Bangladesh.

3 For children >24 months, heights were recorded with children standing; for children <24 months, recumbent height was measured. Heights were recorded to one decimal place.

4 While buffaloes can also produce milk suitable for human consumption, few households in the BIHS sample (∼20) own them. Thus, we do not consider them here.

5 Other survey rounds show a similar distribution.

6 We note two additional results. First, this relationship is non-linear. When we estimate the model including a quadratic term, the linear variable is positive and significant while the quadratic is negative and significant. Second, we assessed the sensitivity of these results by disaggregating by age of child, but the sample sizes become too small to draw meaningful conclusions. Both results are available on request.

7 We note a further feature of these results. Because we have a panel of households, we could also estimate these associations using a household fixed effects specification. But because dairy cow ownership is stable over time (Table 2), differencing over household fixed effects will eliminate much of the variability in cow ownership. Additionally, we lose statistical power as we effectively include 1,169 additional dummy variables – one for each household containing a child in the 12–23.9 month age group. Not surprisingly, when we estimate this model using household fixed effects, we get similar parameter estimates but larger standard errors.

8 See Alderman (1984) and Cunningham (2009).
