# Supplementary material for: Household dairy production, dairy intake, and anthropometric outcomes in rural Bangladesh
Source: Food Policy. 2023 Nov;121:102567. doi: 10.1016/j.foodpol.2023.102567 (PMC10731516; doi:10.1016/j.foodpol.2023.102567)
Supplement: Supplementary data 1 [file mmc1.docx]

**On-line Supplementary Appendix**

For

**Household dairy production, dairy intake, and anthropometric outcomes in rural Bangladesh**

31 October 2023

**Supplementary Table S1: Changes in dairy cow ownership over time**

| Number owned 12 months earlier | Number owned at time of survey | 2011 | 2015 | 2018 |
| --- | --- | --- | --- | --- |
|  |  | Percent | | |
| 0 | 0 (STATIC) | 97.5 | 97.7 | 96.7 |
|  | 1 (ENTRANT) | 2.5 | 2.3 | 3.3 |
|  | TOTAL | 100 | 100 | 100 |
|  |  |  |  |  |
| 1 | 1 (STATIC) | 61.8 | 68.9 | 60.8 |
|  | 0 (EXIT) | 4.7 | 7.3 | 7.4 |
|  | >1 (ACCUMULATE) | 33.5 | 33.8 | 31.8 |
|  | TOTAL | 100 | 100 | 100 |
|  |  |  |  |  |
| 2 | 2 (STATIC) | 54.3 | 55.6 | 53.8 |
|  | 0, 1 (DECUMULATE) | 16.2 | 20.4 | 21.2 |
|  | >2 (ACCUMULATE) | 29.5 | 24.0 | 25.0 |
|  | TOTAL | 100 | 100 | 100 |

**Supplementary Figure S1: Distribution of milk production, conditional on any production, 2018**

**
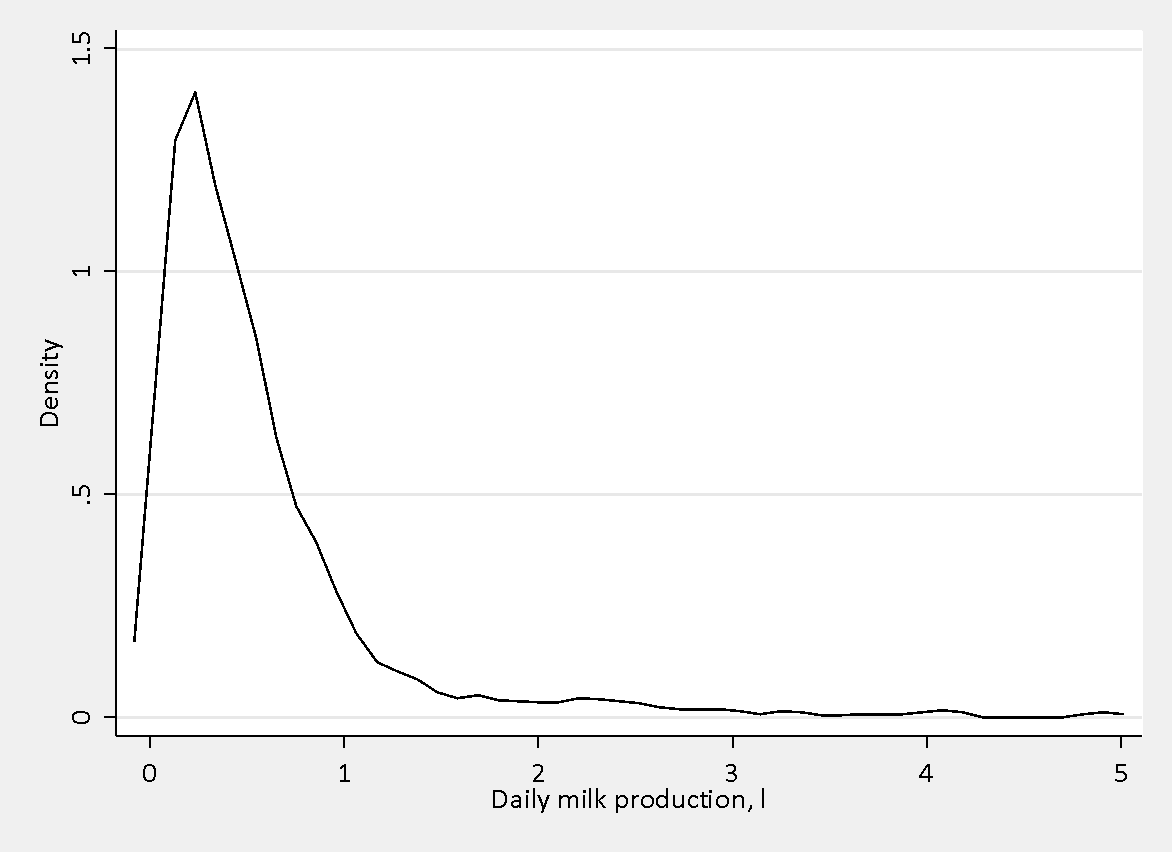
**

Source: Authors’ calculations.
